# Evaluation of Flavonoid Derivative and Doxorubicin Effects in Lung Cancer Cells (A549) Using Differential Pulse Voltammetry Method

**DOI:** 10.15171/apb.2018.072

**Published:** 2018-11-29

**Authors:** Mohammad Amjadi, Jalal Mohammadi Khoshraj, Mir Reza Majidi, Behzad Baradaran, Miguel de la Guardia

**Affiliations:** ^1^Department of Analytical Chemistry, Chemistry Faculty, Tabriz University, Tabriz, Iran.; ^2^Immunology Research Center, Tabriz University of Medical Sciences, Tabriz, Iran.; ^3^Department of Analytical Chemistry, University of Valencia, Valencia, Spain.

**Keywords:** Lung neoplasms, Doxorubicin, Electrochemistry, voltammetry

## Abstract

***Purpose:*** Electrochemical measurements have prompted the progress as a consequence of their affectability, cost-affectivity and comparatively short examination time. The aim of this study was the fast evaluation of the effect of chemotherapy compounds on the viability of lung cancer cells (A549) via electrochemical methods.

***Methods:*** Cyclic voltammetry (CV) was used as a primary method to distinguish between electrochemical behavior of normal and lung cancer cells. Differential pulse voltammetry (DPV) was employed as a complementary analyses method for the impact of doxorubicin (DOX) and Flavonoid modified drug (FMD) (US patent Application number: 62548886) on Lung cancer cells.

***Results:*** Only one oxidative peak, at approximately -0.15 V was detected through DPV method in cancer cell line. While a significant distinguish was not seen in CV. The current intensity (I) was decreased in cancer cells with increasing the DOX and FMD levels (t=99.027, α=0.05, P=0.0000), (t=135.513, α=0.05, P=0.0000), respectively.

***Conclusion:*** The movement of cancerous cells towards death through chemotherapy drugs such as DOX and FMD can make distinct and significant changes in the electrochemical behaviors of those cells.

## Introduction

The intercellular communications, intracellular action and peripheral reactions of cell organelles are as yet not completely comprehended and ensemble averaged measurement of millions of cells together, being not able to give point by point data at single cell level. To evaluate the cell action, single cell analysis can be made by joining capillary electrophoresis with laser induced fluorescence or electrochemical detection, mass spectrometry or flow cytometry.^1^ Electrochemical procedures have prompted the progress in the field of analysis, as a result of their affectability, low cost, and comparatively short examination time, when compared to other methods. Extra utilization of electro-analytical procedures incorporates the assurance of response mechanisms. Redox properties of variety of cells can give some knowledge about its metabolic activity or in vivo redox medication actions.^2^ Differential Pulse voltammetry (DPV) is a form of linear sweep and staircase voltammetry, helpful to assay low levels of biological and mineral analytes in terms of redox activity. In DPV sequences of typical voltage pulses are superimposed on the potential linear sweep or stair steps. Lung cancer cells can be evaluated by DPV method under electrical field and can show the viability of the cells. In the differential pulse voltammogram, the tallness of the current peak obtained as a function of potential can be related to the concentration of analytes. The peak potential shifts being employed to recognize the detected species. DPV also give data about the chemical form of target analytes, as complexation and oxidation status. Hence, this technology has likewise been generally used for the electrochemical examination of proteins and cells.^3,4^

Lung cancer is one of the main sources of cancer related mortality around the world,^5^ being the second most ordinarily recognized malignancy in men and women, that involved approximately 224,390 new cases and 158,080 deaths in 2016 as indicated by the American Cancer Society.^6^ Because of that it is an important point to make an early diagnostics of lung cancer and to evaluate the affectivity of pharmaceuticals developed for its treatment.^7^

Dox is a new class of chemotherapy that can increase the curative of lung cancer cells.^8^ Polyphenols have assigned a special place in the nature in vivo/in vitro studies because of having multiple biological activities, such as: strong anti-oxidants, anti-inflammatory, anti-tumor. Quercetin with the chemical formula (C_15_H_10_O_7_) as one of the important members of the polyphenols group, has the following characteristics:^9^ Free radical scavenger,^10^ inhibitor of TNF-α,^11^ cyclooxygenase 2 and lipoxygenase inhibitors,^12^ and also chelator of intermediate metals such as iron ions that can initiate free radical formation^13^ reducing the expression of a mutated P53 gene in human breast cancer cell line,^12,14,15^ protecting DNA damage from chemicals,^16^ the inhibitor of the synthesis of heat shock proteins,^17^ inhibitor of cyclin D1 and survivin expression,^18^ inhibitor of P21-ras expression in human colon cancer cell line.^19^ The presence of other polyphenols along with quercetin in the FMD (cross linked chitosan-quercetin-other polyphenols) would have an extraordinary synergistic effect in enhancing the antioxidant power. The goal of this study has been the evaluation of A549 line cancer cell electrochemical behavior with carbon electrodes by voltammetry in treatment with FMD and DOX.

## Materials and Methods

A stock solution of 1.0 ×10^-2^ M DOX (Sigma Aldrich, USA) was prepared in ethanol and diluted with water. Phosphate buffer solution (PBS) was used as the medium electrolyte in all experiments. Electrochemical analysis were made using an AUTOLAB 30 analyzer (Eco Chemie Netherlands) with a standard cell of three electrodes; a glassy carbon as working electrode, an Ag/AgCl/3M KCl reference electrode and a platinum auxiliary electrode. The alternating current (AC) voltage amplitude employed was 10 mv and the balance time was 5 s. All tests were made at optimum temperature.^20,21^ Samples were put into the detection cell and handled by DPV in the vicinity of 0.0 and 1.0 V (versus Ag/AgCl) until a stable voltammogram was acquired. Fresh PBS containing living cells was inserted into the measuring chamber immediately. All the electrochemical experiments were done at 37 ± 1°C. The DPV was also be used to quantify the effect of DOX concentration on A549 cell line as follows:

Effect of DOX = 100%× (Ap control – Ap DOX)/ Ap Control being Ap DOX and Ap control the peak areas of DPV obtained for A549 cells treated with and without DOX, respectively.

### Cell examination

Lung cancer cell line A549 and L929, as a normal cell line (Pasteur Institute of Iran) were cultured in RPMI-1640 medium (Sigma Aldrich, USA) with penicillin (100 U/ml), streptomycin (100 μg/ml), FBS 10 % (v/v), and incubated at 37 °C in humidified 5 % CO_2_. After culturing cells for 5 days, they were collected by trypsinization with 0.05 % (v/v) trypsin (Sigma Aldrich), centrifuged at 1200 rpm for 10 min, and then suspended in PBS. The concentration of the cells was examined by standard pour plate (PPC) technique. For drug tests, cells were admitted to proliferate and stick for 24 h in culture medium before exposure to DOX. A similar amount of alcohol was supplemented to the control cells to exclude the impact of ethanol on the lung cancer cells. Morphological changes of the cells, before and afterwards of the presence to DOX, were seen with a Nikon Eclipse optical microscope TE-300 (Japan). Cell viability was checked by the 3- (4, 5-dimethyl thiazol-2-yl)-2, 5-diphenyl-2H tetrazolium bromide (MTT) colorimetric assay. Cells (15×10^3^ cells/well in 96 wells) were handled with various concentrations of DOX (0-600 μg/mL) for 24 hours, then 0.5 mg/mL in dimethyl sulfoxide (DMSO) was added to each well. After incubation at 37 °C for two hours, an equal volume of 90 % isopropanol and 0.5 % sodium dodecyl sulfate (SDS) (400 μL) was added to dissolve the MTT formazan crystals, and the absorbance was measured at 570 nm using an ELISA microplate reader (State Fax 2100, USA). The percentage of cell viability was calculated as: (A549 of treated cells/ A549 of untreated cells) ×100. A549 cell viability was accomplished at the same time by treating the cells with different concentrations of DOX.^22^

### Statistical analysis

The data were subjected to standard one-way analysis of variance (ANOVA). The means were compared using Duncan Multiple Range test at the 5% confidence level also t-test was used to test the statistical difference between two group means at the 5% confidence level. Data was analyzed in SPSS19 and MSTATC software.

## Results and Discussion

### Electrochemistry examinations 

**Figure 1:**
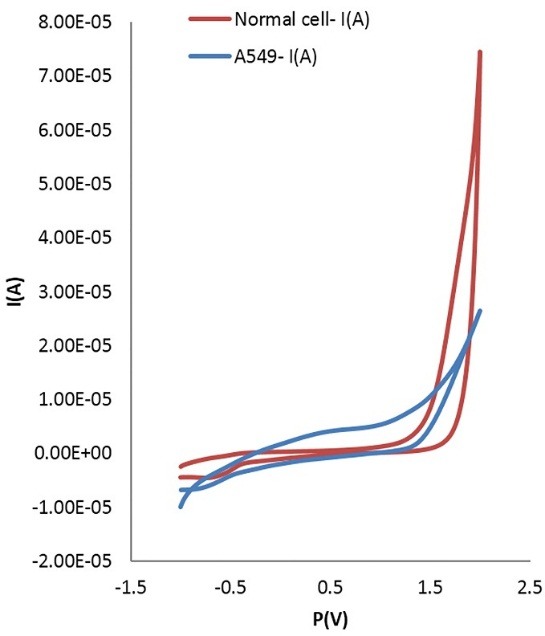
Comparison between CV results of lung cancer cells and normal cells in PBS at glassy carbon electrode; for the oxidative peak (upper line) and the reductive peak (lower line). Cell density, 3×10^6^ cells mL^–1^; scan rate, 50 mV s^–1^. (t=0.677: α=0.05, P=0.5)

**Figure 2:**
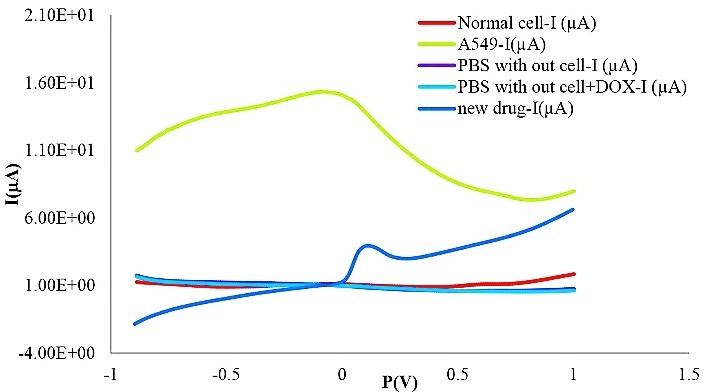
Comparison between DPV responses of lung cancer cells and normal cells in PBS and presence of FMD. Cell density: 3×10^6^ cells mL^–1^; scan rate, 50 mV s^–1^.

Comparison between cyclic voltammetry (CV) outcomes of lung cancer and normal cells in PBS are illustrated in Figure 1 at a scan rate of 50 mV s^–1^, while reductive peak was detected under the same position. In Figure 2 an apparent peak around -0.15 V was detected when the cells were added to the PBS. No peak was found in the blank solution, normal cell line and also PBS solution. The mean difference between the various groups along with normal cell line was significant in comparison with A549 (F=3.227 E15, α=0.05, P=0.0004). However, this difference was not statistically significant among all groups except A549.

**Figure 3:**
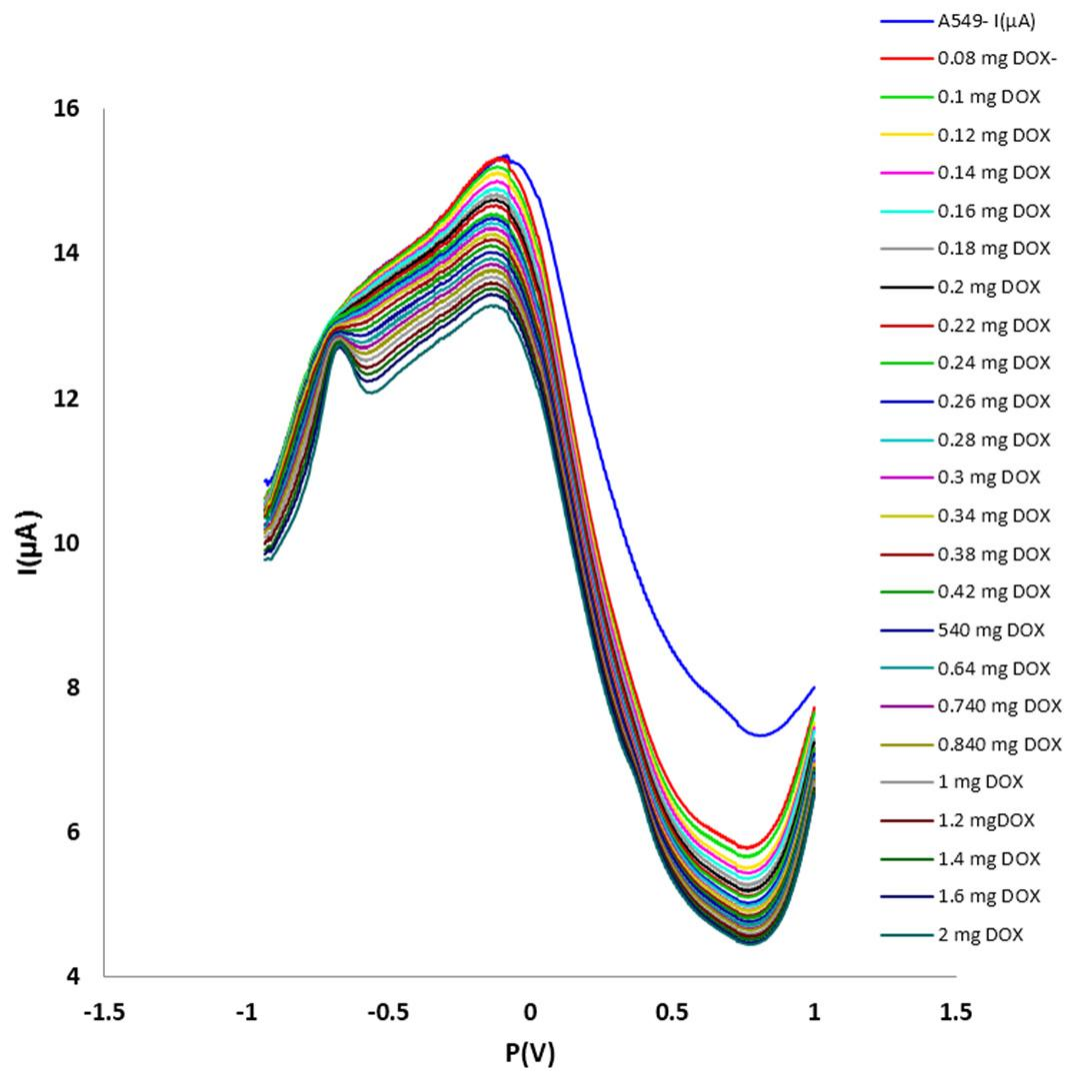
Effect of different concentrations of DOX on lung cancer cells A549. Cell density: 3×10^6^ cells mL^–1^; scan rate, 50 mV s^–1^.

**Figure 4:**
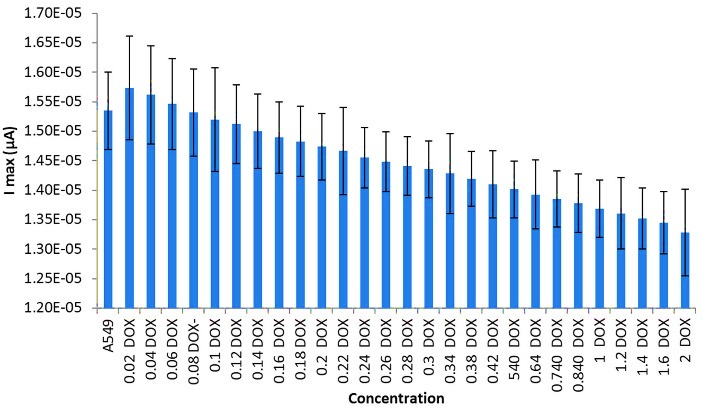
Effect of different concentrations of DOX on lung cancer cells A549.

**Figure 5:**
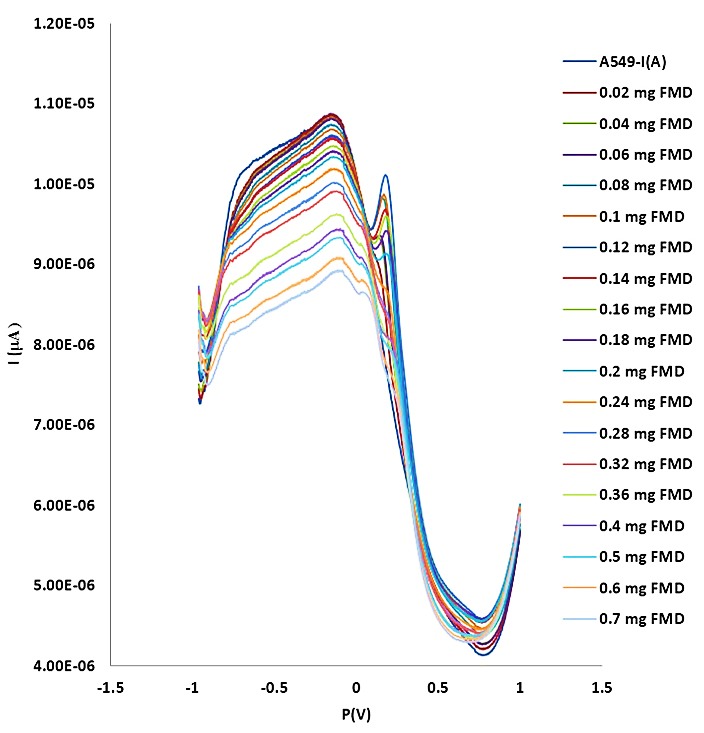
Effect of different concentrations of FMD on lung cancer cells. Cell density: 3×10^6^ cells mL^–1^; scan rate, 50 mV s^–1^.

**Figure 6:**
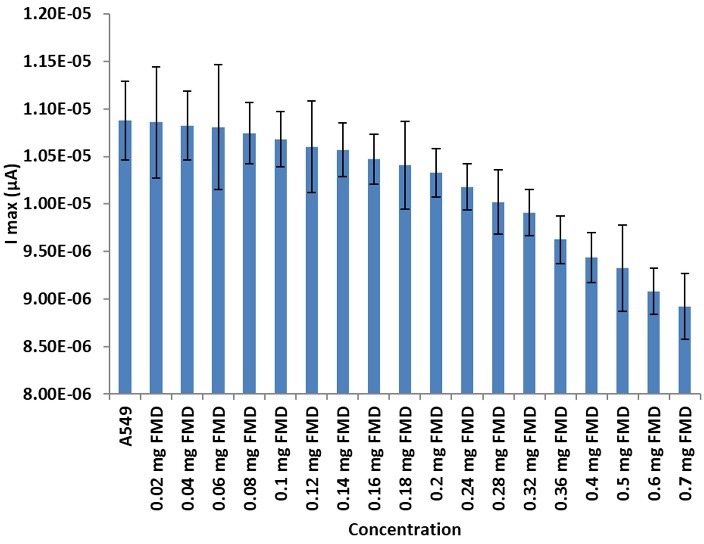
Effect of different concentrations of FMD on lung cancer cells A549. I max based on mg FMD

Figure 3 and 4 show the DPV method of different concentrations of DOX on the A549 cell line. For DOX concentration from 0.02to 0.08 mg, the I index was decreased (t=99.027, α=0.05, P=0.0000). It needs to be mentioned that the I index was as same as A549 cell line around 0.08 mg. No crucial difference was seen when the concentration of DOX was higher than 100 μM. From 0.08 to 2 mg, the rate of decreasing was continued. Figure 5 and 6 show the impact of various concentrations of FMD on lung cancer cells which A549 cell line without any drug had the highest I (10.9 µA) among different concentration of FMD (t=135.513, α=0.05, P=0.0000). As FMD concentration was increased the I index decreased regularly up to 8.92µA.

Electrochemical techniques have been confirmed as a sensitive way for detection of biological molecules.^23^ With simple, rapid, sensitive and easily operated features, DPV has been applied to evaluate cell viability in the presence of pharmaceuticals.^4^ The electrochemical behavior of A549 cancer cell line in relation with DOX and FMD at glassy carbon electrode was assembled and examined for the first-time through this study. Feng *et al*. studied electrochemical behavior of U937 cells and its performance in evaluating the effect of caffeic acid. They exploited the electrochemical procedure to investigate the effectiveness of caffeic acid on the treatment of cancer cells. This finding was parallel to the hindrance effect of caffeic acid on the viability of U937 explored by the trypan blue test,^24^ which are also in agreement with the results of this study.

In a study of Chunmei Yu *et al*‏.^25^ by modified electrode on K562 cancer cell line treated with arsenic trioxide and cyclophosphamide through CV and DPV exhibited that increasing the drug concentration induced the decrease of electrochemical responses significantly. In another works by João G. Pacheco et al‏. and Kun Wang et al‏.,^26,27^ the preparation of a new biosensor with modified electrodes for breast cancer cells and the use of CV-DPV electrochemical methods to determine the amount and number of cancer cells by plotting the calibration curve allowed the detection of the number of cancer cells in the unknown sample.

In 2005 the electrochemical action of breast tumor cells (MCF-7) was assessed by CV and potentiometric stripping analysis (PSA) by Li and co-workers.^3^ The MCF-7 cells were found to generate a PSA signal. The influence of diosgenin on the MCF-7 cells was also successfully evaluated by PSA. In the present study, CV was used for introductory experiments for distinguishing normal and lung cancer cells. Because of its high efficiency, DPV was used for complementary survey of the effect of pharmaceuticals on cell lines viability. The peaks obtained from electrochemical analyses of normal and cancer cells were different. Methods as MTT were already employed for exploiting the effects of medication on cancer cells. The effects of drugs on normal and cancer cells were inspected by the electrochemistry measurements, including CV and DPV. As shown in Figure 3, at concentrations of approximately 0.08 mg of DOX, it may be able to activate the potential pathway of apoptosis.^28^ There is a peak around -0.5 V, and that is related to the DOX drug.^29^

Rising redox in the estimated limit of -0.15V in A549 cancer cell line seems to be due to the formation of pyruvate and lactate in the context of the Warburg phenomenon. The impact of doxorubicin dose providing a strong reduction of carcinoma cells, and as a result, we could be faced with the probable decline of pyruvate and lactate.^30-32^ However, the use of DPV requires about one minute, while MTT test needs about 7 days to be finished. The FMD drug showed better results in cell death compared to DOX, while both drugs standard concentration were the same. For probably apoptosis of 3 million cells, a dose of 2 mg of DOX was required, but for FMD only 0.7 mg was used.^33^

## Conclusion

In this study, the effects of two different drugs on cancer cells were investigated through CV and DPV. Results show that FMD (US patent Application number: 62548886) to treat the cancer, has a much better effect than DOX. Additionally, since in this survey unmodified carbon glassy electrode was exploited, this technique can be considered highly reproducible and could be used in clinical trials.

## Ethical Issues

Not applicable.

## Conflict of Interest

The authors declare that they have no conflicts of interest.
